# P-1703. How Low Should We Go?: Experience Using a Threshold of 1,000 CFU/ml for Reporting Uropathogens from Midstream Urine Samples

**DOI:** 10.1093/ofid/ofaf695.1875

**Published:** 2026-01-11

**Authors:** Shelby Pandy Willis, Andrea H Son, Michelle T Hecker

**Affiliations:** MetroHealth Medical Center, Cleveland, OH; The MetroHealth System, Cleveland, Ohio; The MetroHealth System, Cleveland, Ohio

## Abstract

**Background:**

Women with symptomatic uncomplicated urinary tract infections (UTI) may have urine cultures with uropathogens isolated at levels of growth as low as 100 CFU/ml. The American Society for Microbiology suggests a threshold of ≥ 100,000 CFU/ml for reporting growth of uropathogens from midstream urine samples but recommends reporting any amount of *Streptococcus agalactiae* from women of child-bearing age. Reporting thresholds vary across institutions. Our lab uses a reporting threshold of ≥ 1000 CFU/ml for all midstream urine cultures. Herein we describe the characteristics and management of patients with low colony count (1,000 – 10,000 CFU/ml) urine culture results.

**Table 1:**
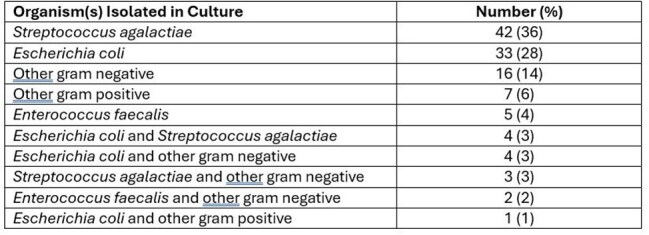
Organisms isolated in culture

**Table 2:**
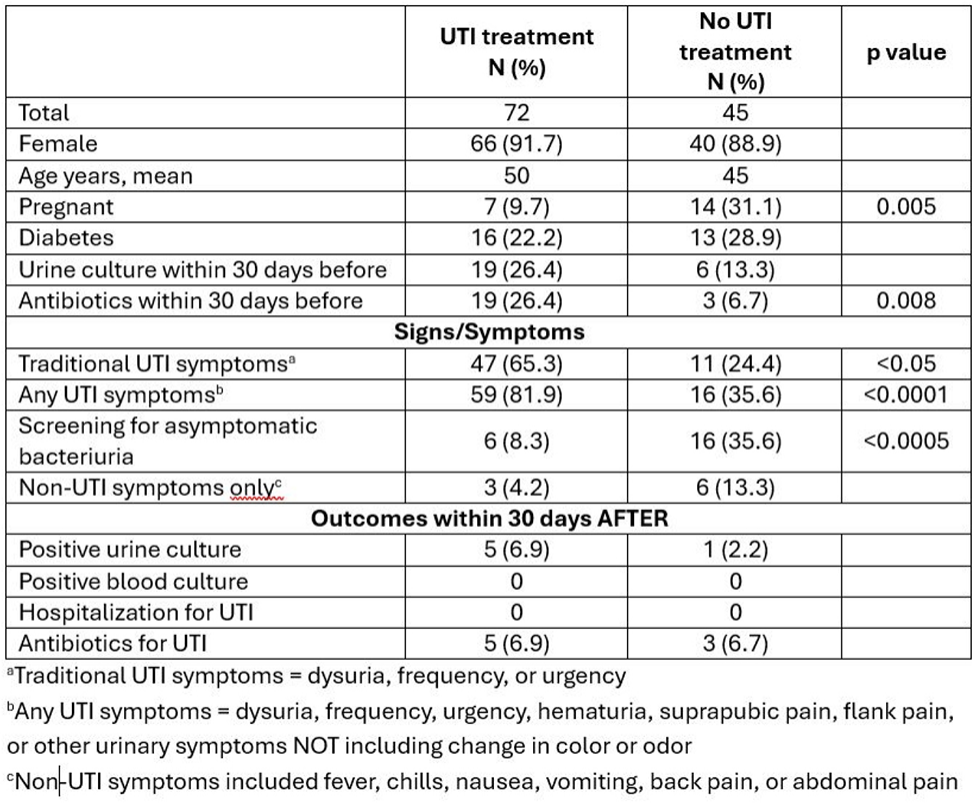
Comparison of those prescribed antibiotics for UTI vs. those not prescribed antibiotics for UTI

**Methods:**

Retrospective chart review of adult outpatients with a midstream urine culture with 1 or 2 uropathogens at a level of growth between 1,000 and 10,000 CFU/ml from 1/1/2024 - 12/31/2024. Approximately 10 unique patients were randomly selected from each month.

**Results:**

Of 117 patients reviewed, 106 (91%) were female with an average age of 48 years. *Streptococcus agalactiae* and *Escherichia coli* were the most common organisms reported (Table 1). Antibiotics for UTI were prescribed for 72 (61.5%) patients. Those who received UTI treatment were more likely to have had antibiotics within the 30 days prior and have urinary tract symptoms. Those who did not receive UTI treatment were more likely to be pregnant and to have had urine culture done for asymptomatic bacteriuria screening (Table 2). Of those who received UTI treatment, 59 (82%) had one or more UTI related symptoms, 3 of whom also tested positive for a genital tract infection (2 *Herpes simplex*, 1 *Trichomonas vaginalis*). Six (8%) had no symptoms but were being screened for asymptomatic bacteriuria, and 7 (10%) had nonspecific symptoms without any UTI related symptoms.

**Conclusion:**

: Over 75% of persons treated for a positive urine culture with low colony counts had symptoms potentially attributable to a UTI without a positive genital tract test. These patients may have been misdiagnosed as not having a UTI if the urine culture was not reported as positive. Approximately 20% of patients may have received unnecessary UTI treatment for low colony count asymptomatic bacteriuria and non-UTI syndromes.

**Disclosures:**

All Authors: No reported disclosures

